# Patients and Spiders

**Published:** 1980

**Authors:** Philip Radford

**Affiliations:** General Practitioner, Westbury-on-Trym, Bristol


### Patients and Spiders

**Philip Radford**
General Practitioner, Westbury-on-Trym, Bristol

Once, while in country practice, I was asked to see an inexperienced farm worker who had lacerated a leg by the over-zealous use of a scythe; the telephone message stated that the wound was bleeding badly. On arrival at the farm, however, I found no panic and, on inspecting the deep wound, I found very little blood. The laceration had been packed with cobwebs, which had been pulled hastily from corners of the cowshed; the intricate mesh of multiple, fine silken threads had produced excellent haemostasis.

Of course, this procedure was a long-established country method of first-aid. Cobwebs have been used as applications to bleeding wounds for many centuries in rural areas; no doubt battle casualties, in the Middle Ages, for example, were often treated in this fashion.

The patient I saw at the farm made a good recovery, following initial suturing. Tetanus did not supervene and no local sepsis was observed. Yet, it is obvious that bacteriological investigation of stable and pigsty spiders' webs is not going to yield results to satisfy the modern microbiologist. Doubtless it is simpler, and certainly far safer, in a case of extensive capillary haemorrhage, to apply pressure to the wound by means of the usual sterile gauze pad. But there might still be the odd occasion when spiders' webs are the only suitable dressing material available in an emergency.

Cobwebs are not welcomed in many households today, even though they may be tolerated in the stable. On the discovery of a spider's web the housewife usually sweeps it away, to be deposited in the dust-bin. Sometimes the dislike of webs is extended to the spider itself; many people flee from spiders and some become intent on killing them. Such persons maintain that the quick and unexpected movement of these eight-legged creatures is really upsetting to them; usually it is women who hold these views.

Clearly, Miss Muffet's behaviour and influence continues at the present day. Some time back I interviewed an intelligent woman who told me that she had been accused of shoplifting. She explained that she had been scared that day by a big, black spider which had run suddenly across the floor of a shed; apparently, she had been so upset by the incident that on going into a store that afternoon she had not been responsible for her actions. Having told me the story, she said that the expression on my face had shown that I had not believed her and, because of my reaction, she felt that she could not possibly give such an explanation before a court of law. In her opinion, doctors just did not understand how terrified some people were of spiders and she seemed to think that magistrates had a similar lack of sympathy. Rightly or wrongly, I let the matter rest there.

Women are not alone in being afraid of spiders. I recall a male patient who flatly refused to visit Burma on the grounds of his intense spider-phobia although, to be fair, he also had a fear of snakes and scorpions. Perhaps I should have delved deeply into psychological events of his childhood to see if some relief could be given but, as life in Britain was satisfactory for him, I did not initiate any investigation of his mental state. Happily, he was reassured that none of Britain's numerous spider species could inflict physical harm on man. Even so, I understand that he continued to flee from the presence of spiders.

Whatever may be our attitude to moving spiders, or spiders suddenly located in some cranny, most of us are prepared to admire the beauty of spiders' webs. Some autumn scenes are made lovely by gossamer, composed of the numerous webs of so many small spiders; an October afternoon may well reveal such a multitude, borne on the herbage or floating in the air if sufficiently dry.

A fine description of a fall of gossamer was given by the naturalist Gilbert White in the 18th Century; in one of his letters in *The Natural History of Selborne* he tells how a shower of cobwebs continued for the whole of one day in late September and, had anyone wished, whole baskets of cobwebs could easily have been gathered.

For small spiders to produce such an amount of fine, silk fibre is surely a remarkable and specialised process. The silk threads are prepared by the actions of the two or three pairs of spinning organs situated at the back of the abdomen of the spider; these spinnerets have multiple spinning tubes from which the silk, coming from the silk glands, is extruded.

It is not surprising that many birds make use of cobweb, building this light and filmy material into their nest structures, thereby adding to the insulation and giving decoration and camouflage.

Probably the best-known cobweb architect is the long-tailed tit *Aegithalos caudatus;* with this bird webs are interwoven with lichens and wool to make a cosy, ovoid nest which, with a lining of hundreds of feathers, must give as much comfort and warmth to the nestlings as is possible in so small a space.

Another common bird to seek spiders' webs for nest construction is the chaffinch *Fringilla coelebs.* In this case, web material is twined with moss, grasses and lichens; the resulting inconspicuous nest blends beautifully with a lichened tree branch. Then goldfinches *Carduelis carduelis,* building on branches of orchard trees, mix rootlets with the wool of sheep and spiders' webs; hence the young find a well-insulated and strong nest-base when hatched from their eggs. Yet another bird skilful in incorporating spiders' webs into the wall of its nest is the goldcrest *Regulus regulus.* Here, the suspended, bag-type nest is normally composed of mosses and webs.

We have no information as to whether Miss Muffet approved of the design of spiders' webs even though she disliked their builders. Nevertheless, many persons enjoy the sight of webs outlined by frozen dew on a December morning and admire the geometric pattern of the concentrically placed and radial silk fibres; such webs look particularly attractive when stretched between the dead, frosted stems and the seed-heads of umbelliferous plants such as hogweed.

We admire also the agility of the spider in grasping the fine, silk lines of its web and note the speed with which it rushes to deal with an entrapped fly. But perhaps most of all we delight in watching a spider descend to the ground by its ever-lengthening, thin silken thread, prepared as it travels, and wonder at the mechanism of its production. With such an activity before one, it is surely difficult to follow the cult and example of Miss Muffet.

While many British bird species utilise spiders' webs regularly in the building of their nests, others search for spiders as a normal article of diet. The tiny wren *Troglodytes troglodytes,* for instance, ingests a remarkable number of spiders in the course of a day, often including some remarkably large specimens. Goldcrests, the smallest of British birds, behave in the same way, looking diligently in bark crevices for spiders which form a high proportion of their food intake. One can but speculate whether any such birds behave like Miss Muffet and, if so, do any of them survive a truly hard winter?

Just as many birds eat spiders, so there are some large, tropical spider species which swallow small birds. Birds living in the vicinity of these spiders obviously have to be vigilant and, to survive, it behoves them to follow the behaviour of Miss Muffet. Vulnerable birds could well be excused for developing a spider-phobia and, indeed, their continued existance depends on its development and constant maintenance. Yet why should certain humans, who have nothing to fear from spiders and who have the mental ability to comprehend their skilled and characteristic activities, fear them so strongly? It is a pity that Miss Muffet does not live today, so that we might have a full psychiatric report on her background and present symptoms. Perhaps an effective treatment could even be considered for her.

